# MRI-based radiomics models to assess prostate cancer, extracapsular extension and positive surgical margins

**DOI:** 10.1186/s40644-021-00414-6

**Published:** 2021-07-05

**Authors:** Dong He, Ximing Wang, Chenchao Fu, Xuedong Wei, Jie Bao, Xuefu Ji, Honglin Bai, Wei Xia, Xin Gao, Yuhua Huang, Jianquan Hou

**Affiliations:** 1 Department of Urology, The First Affiliated Hospital of SooChow University, No.188, Shizi St, Canglang District, 215006 Suzhou, Jiangsu China; 2 Department of Radiology, The First Affiliated Hospital of SooChow University, No.188, Shizi St, Canglang District, 215006 Suzhou, Jiangsu China; 3 Suzhou Institute of Biomedical Engineering and Technology, Chinese Academy of Sciences, No.88 Keling Road, Suzhou New District, 215163 Jiangsu, China; 4 The School of Electro-Optical Engineering, Changchun University of Science and Technology, 130013 Changchun, China; 5 Department of Urology, Dushu Lake Hospital affiliated to SooChow University, No.9, Chongwen Road, Suzhou Industrial Park District, Suzhou, Jiangsu 215000 China

**Keywords:** Prostate cancer, Radiomics, Extracapsular extension, Positive surgical margins

## Abstract

**Purpose:**

To investigate the performance of magnetic resonance imaging (MRI)-based radiomics models for benign and malignant prostate lesion discrimination and extracapsular extension (ECE) and positive surgical margins (PSM) prediction.

**Methods and materials:**

In total, 459 patients who underwent multiparametric MRI (mpMRI) before prostate biopsy were included. Radiomic features were extracted from both T2-weighted imaging (T2WI) and the apparent diffusion coefficient (ADC). Patients were divided into different training sets and testing sets for different targets according to a ratio of 7:3. Radiomics signatures were built using radiomic features on the training set, and integrated models were built by adding clinical characteristics. The areas under the receiver operating characteristic curves (AUCs) were calculated to assess the classification performance on the testing sets.

**Results:**

The radiomics signatures for benign and malignant lesion discrimination achieved AUCs of 0.775 (T2WI), 0.863 (ADC) and 0.855 (ADC + T2WI). The corresponding integrated models improved the AUC to 0.851/0.912/0.905, respectively. The radiomics signatures for ECE achieved the highest AUC of 0.625 (ADC), and the corresponding integrated model achieved the highest AUC (0.728). The radiomics signatures for PSM prediction achieved AUCs of 0.614 (T2WI) and 0.733 (ADC). The corresponding integrated models reached AUCs of 0.680 and 0.766, respectively.

**Conclusions:**

The MRI-based radiomics models, which took advantage of radiomic features on ADC and T2WI scans, showed good performance in discriminating benign and malignant prostate lesions and predicting ECE and PSM. Combining radiomics signatures and clinical factors enhanced the performance of the models, which may contribute to clinical diagnosis and treatment.

**Supplementary Information:**

The online version contains supplementary material available at 10.1186/s40644-021-00414-6.

## Introduction

Prostate cancer (PCa) is the second most common cancer in males worldwide [1]. According to the most recent cancer statistics estimated by the American Cancer Society, PCa alone accounted for nearly 20 % of new cancer diagnoses and 10 % of cancer deaths in males in 2019 [2].

The clinical gold standard for PCa diagnosis is prostate biopsy, but biopsy may lead to complications such as pain, bleeding, inflammation and dysuria [3–5]. Prostate-specific antigen (PSA) tests and digital rectal examinations (DREs) are widely used as non-invasive methods to detect PCa [6]. PSA tests and DREs have high sensitivity but low specificity [7].

After PCa is detected, staging is an important task that significantly influences management of the disease. The evaluation of extracapsular extension (ECE), which indicates that PCa has reached stage T_3_, is of significance because ECE is associated with cancer-specific survival and can affect the positive surgical margins (PSM) [8]. PSM is regarded as a negative prognostic factor in PCa patients [9]. The presence of PSM within a radical prostatectomy (RP) specimen has a negative effect on prognosis and is linked to a 3.7-fold increase in the risk of biochemical recurrence [10].

To improve the risk assessment of ECE and PSM, many nomograms based on PSA, age, perineural invasion status, Gleason score and percentage of positive cores in biopsy pathology have been constructed [9, 11, 12]. However, ECE and PSM are still poorly predicted by standard clinical tests.

Multiparametric MRI (mpMRI) is considered a standard tool for diagnostic evaluations of PCa and can help reduce unnecessary biopsies by a quarter [13, 14]. The accurate assessment of PCa by mpMRI before RP can help clinicians distinguish extraprostatic disease, identify risk factors associated with PSM, and evaluate intraoperative complications and functional recovery after surgery [15]. Several studies have demonstrated that the features extracted from T2WI and ADC can aid the classification of Gleason scores [16–19], indicating that the association between extraprostatic disease and the features extracted from ADC and T2WI can be used to improve the accuracy of benign and malignant prostate lesion discrimination and ECE and PSM prediction.

Radiomics is a novel tool that can translate images into meaningful data for analysis and has been applied in oncology and the development of machine learning methods [20]. Recent studies have explored the value of radiomics based on MRI in the differentiation of PCa from benign prostate tissue [21] and the evaluation of PCa aggressiveness [22]. Additional applications of radiomics are possible.

In this study, we attempted to investigate whether an MRI-based radiomics model could aid clinical diagnosis and treatment due to its efficiency in benign and malignant prostate lesion discrimination, ECE prediction and PSM prediction.

## Materials and methods

### Patients and data collection

With the approval of our institutional review board, we performed a retrospective analysis of 640 consecutive patients who underwent pelvic mpMRI and prostate biopsy at the First Affiliated Hospital of SooChow University.

Patients who met the following inclusion criteria were included in our study: (1) prostate lesions with well-defined boundaries on both T2WI and ADC images according to the Prostate Imaging Reporting and Data System version 2 (PI-RADS v2); (2) clinical information, including age, total PSA (tPSA), free PSA/tPSA (f/tPSA), biopsy Gleason score (biopsyGS), and percentage of positive cores; (3) mpMRI before biopsy; (4) trans-rectal ultrasound guided random biopsy including at least 12 cores; and (5) RP performed in PCa patients by the same urologist within three months after MRI and biopsy.

The exclusion criteria were as follows: (1) previous prostate biopsy; (2) previous treatment for PCa; and (3) confirmed diagnosis of a tumor other than PCa.

The following three biological characteristics were studied using the above patient data: discrimination of benign and malignant prostate lesions in all patients and ECE prediction and PSM prediction in patients with malignant prostate lesions.

### Magnetic resonance imaging protocols

All patients were scanned with a 3.0T MRI scanner (MAGNETOM Skyra; Siemens Healthineers, Erlangen, Germany) using a standard spine array coil and an 18-channel body array coil. The images included axial, coronal and sagittal T2WI (repetition time (TR)/echo time (TE), 3900/105 ms; flip angle, 160°; section thickness, 3 mm; intersection gap, 0 mm; FOV, 25 cm; and matrix, 384*336) and axial DWI (diffusion weighted imaging) (b values, 0, 700, 1400, and 2000 s/mm^2^; TR/TE, 5000/72 ms; section thickness, 5 mm; FOV, 20 cm; and matrix, 128*128). ADC was obtained from DWI with b values of 0 and 700 s/mm^2^ using a 2D echo planar imaging sequence.

### Pathological Evaluation

After the biopsy or the prostatectomy, the excised tissue was submitted to histopathology. The RP specimen was fixed in formalin and sliced from apex to base in 3 to 4 mm intervals. These slices were then stained with haematoxylin and eosin (H&E). A pathologist with 5 years of experience outlined each lesion on the microscopic slices and assigned a Gleason score. ECE was determined by the presence of neoplastic tissue outside the prostatic capsular in the periprostatic tissue. PSM was determined by the presence of neoplastic tissue at the surgical margins. Another radiologist with over 5 years cooperated with the pathologist on the pathology-MRI lesion matching. All the results were confirmed and corrected by the other pathologist and radiologist both with over 15 years of experience.

### Radiomic feature extraction

**Fig. 1:**
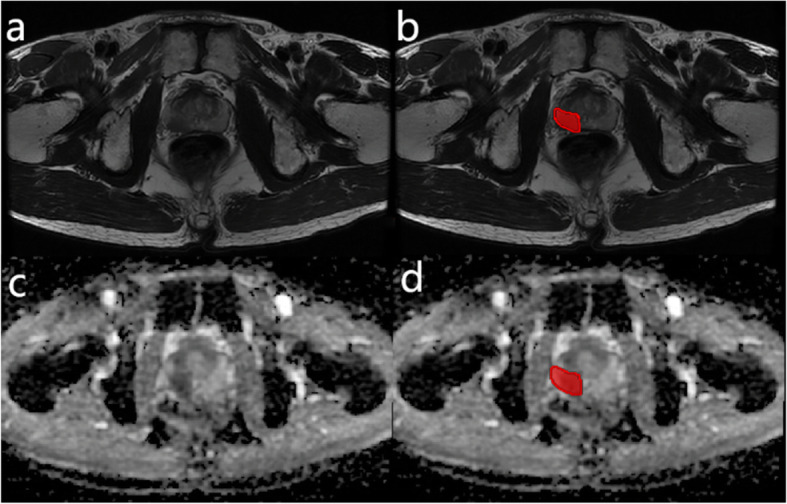
Examples of ROI delineation. **a** and **c** are T2WI and ADC images, respectively, of the same patient. The red areas in **b** and **d** were delineated as ROIs

Experienced pathologists and radiologists reached a consensus regarding the standard histological-radiological correlation based on the histological and imaging findings. The tumor region of interest (ROI) was identified by anatomical landmarks and manually delineated slice by slice on both the T2WI and ADC sequences by radiologists using Medical Imaging Interaction Toolkit (MITK) software (version 2013.12.00) (Fig. 1). The full intensity range of the ROI was quantized to 32 Gy levels for subsequent feature extraction.

The radiomic features were extracted from the ROIs on both the T2WI and ADC sequences of each patient by using the open-source Python package Pyradiomics (version 2.1.0) [23]. The extracted features were divided into the following three categories: (1) 14 shape-based features, including descriptors of the three-dimensional size and shape of the ROI; (2) eighteen first-order features describing the distribution of the voxel intensities within the ROI using commonly used and basic metrics; and (3) 75 texture features, including the gray-level run-length matrix (GLRLM), gray-level co-occurrence matrix (GLCM), gray-level size-zone matrix (GLSZM), neighboring gray-tone difference matrix (NGTDM) and gray-level dependence matrix (GLDM). The wavelet features were calculated by wavelet decomposition to obtain the intensity and texture features of the original image. The features were concentrated in different frequency ranges within the tumor ROI [24]. In this research, all radiomic features, except for the shape-based features, were calculated based on the original image and derived image obtained by applying a wavelet transform. Ultimately, 851 quantitative radiomic features were extracted for each ROI.

### Feature selection

The number of radiomic features was much larger than the number of patients, and feature selection was performed to avoid overfitting. A portion of the features may have low reproducibility when the ROI is manually delineated by different radiologists or at different times. To eliminate these features with low reproducibility, two radiologists (radiologist 1 with 6 years of experience in MRI interpretation and radiologist 2 with 10 years of experience) were assigned to delineate the ROIs in each case. Radiologist 1 performed a series of delineations at two different times, and radiologist 2 performed the delineation only once. The interclass and intraclass correlation coefficients (ICCs) were computed to assess the interobserver (radiologist 1 vs. radiologist 2) and intra-observer (radiologist 1) reliability, respectively. A large ICC value represents a high degree of reproducibility. Features with an ICC lower than 0.75 were considered to have poor agreement and were removed.

Features with a variance close to 0 were redundant and could not provide valid information for predicting the label because the values of such features barely change regardless of whether the case is negative or positive. A Spearman correlation analysis was performed to identify the highly correlated features [20]. Features with a mean absolute correlation higher than 0.9 were considered redundant. Standardization was performed to eliminate the impact of different feature orders of magnitude by scaling the features to a zero mean and unit variance.

Previous studies have shown that adding a prior feature ranking procedure may be helpful in improving the final performance. After eliminating the features with low reproducibility or high redundancy, we used feature-ranking algorithms to identify the most important features for label prediction based on a heuristic scoring criterion, and only the top-ranked features were retained. The minimum redundancy, maximum relevance (MRMR) approach is a representative and highly cited multivariate ranking method [25]. This method can find the *m* features most relevant to the label from the feature space.

### Model building

The least absolute shrinkage and selection operator (LASSO) regression algorithm was implemented to explore powerful predictive combinations of features related to labels and reduce overfitting and selection bias. We used the LASSO classifier to build radiomics signatures based on the top-ranking features of each single sequence and a multivariable logistic regression to build mpMRI signatures by combining predictions of different radiomics signatures. The classifier was trained using 10-fold cross-validation and the training set.

To assess the impact of the clinical parameters, we built integrated models by adding the clinical characteristics to the radiomics signatures. We used independent t-tests or Mann-Whitney U tests to assess the continuous variables and chi-square tests to evaluate the categorical variables. The optimal model was chosen from combinations of radiomics signatures and subsets of clinical characteristics by using a multivariable logistic regression analysis and Akaike information criteria (AICs) [26].

The radiomics signatures and integrated models were tested using an independent testing set. The areas under the receiver operating characteristic (ROC) curves (AUCs) along with the 95 % confidence intervals (CIs) and accuracy values were calculated to assess the classification performance, and the cut-off value was selected according to the Youden index to determine the corresponding sensitivity and specificity. The DeLong test [27] was used for the statistical comparison of the AUCs between the integrated models and the corresponding radiomics signatures.

The radiomics signature extracted from T2WI is denoted by ${S}_{T2WI}$, the signature extracted from ADC is denoted by ${S}_{ADC}$, and the signature of the combination of features extracted from T2WI and ADC is denoted by ${S}_{MP-MRI}$. The integrated diagnosis models are correspondingly denoted by ${M}_{T2WI}$, ${M}_{ADC}$ and ${M}_{MP-MRI}$.

R software (3.6.1) was used to conduct feature selection, model building, and statistical analysis.

## Results

### Patient profiles

**Fig. 2:**
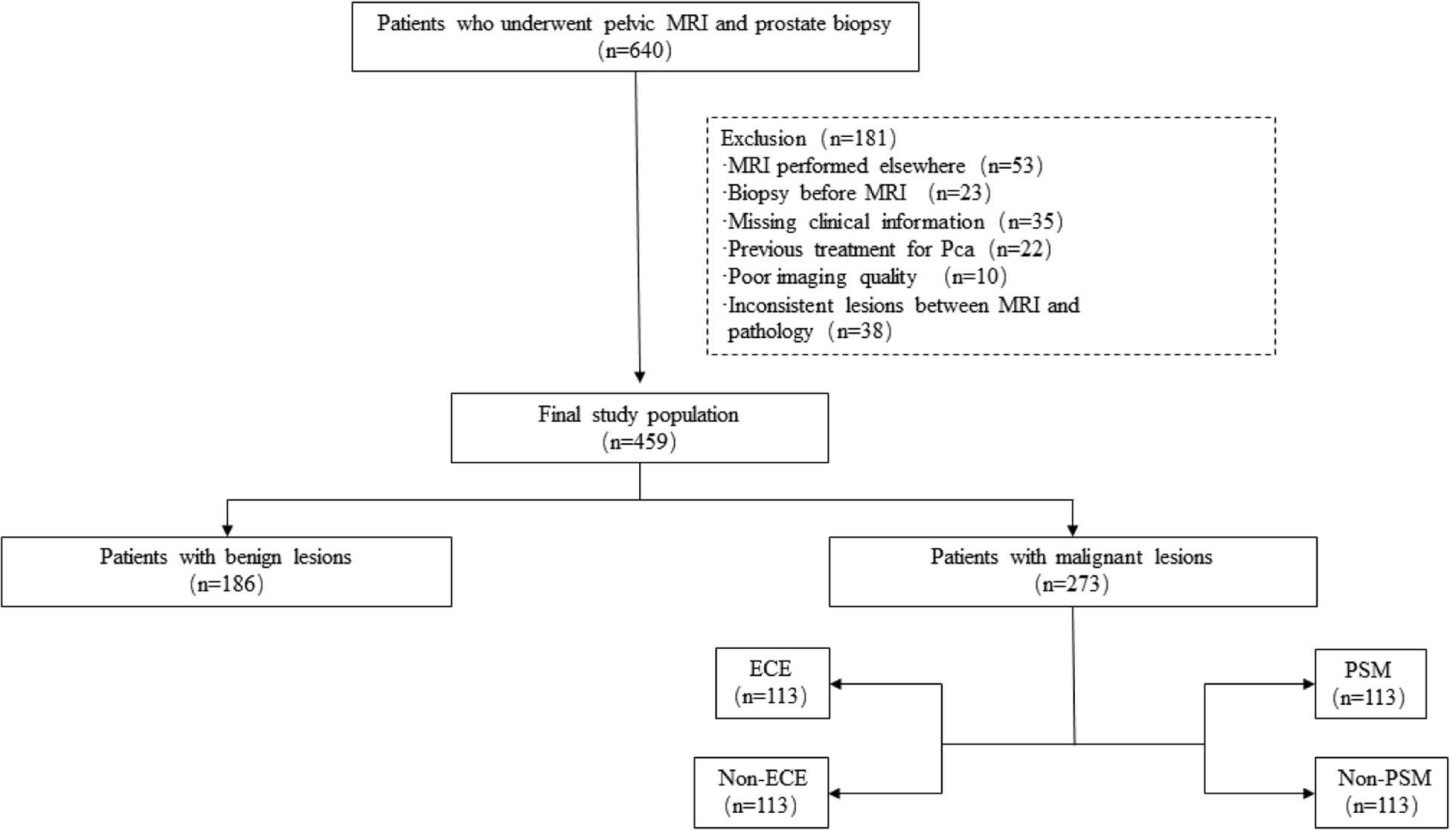
Flow diagram of patient selection for the study

In total, 459 patients with prostate disease (186 patients with benign tumors and 273 patients with malignant tumors) were analysed after screening according to the inclusion criteria (Fig. 2). 186 patients were diagnosed of benign diseases through biopsy and the other 273 patients with malignant tumors all underwent prostatectomy. The data included clinical information (age, tPSA, f/tPSA, positive core percentage, biopsyGS, MRI report and postoperative pathology report; Table 1) and pelvic mpMRI images. A stratified sampling method was used to divide the data into the training set and testing set at a ratio of 7:3. Of the 459 patients in this study, 323 were assigned to the training set, and 136 were assigned to the testing set. The ECE and PSM were studied using the 273 patients with malignant tumors, and the training and testing sets for these two labels contained 192 patients and 81 patients, respectively.

**Table 1:** Clinical information of patients with benign and malignant prostate disease

	Benign	Malignant
Number	186	273
Age, mean (range) (year)	65.23 (30–89)	70.21 (49–87)
tPSA (ng/ml), mean (range)	13.62 (1.64-136.45)	28.95 (2.85-211.87)
f/tPSA, mean (range)	0.16 (0.03–0.40)	0.13 (0.02–0.43)
PI-RADS v2, median (range)	3 (2–5)	4 (3–5)
Positive core percentage, mean (range)	-	0.41 (0.08-1)
BiopsyGS, median (range)	-	7 (6–10)
ECE reported by MRI	-	25
ECE on postoperative pathology, n	-	113
reported by MRI, n (%)	-	23 (20.4)
not reported by MRI, n (%)	-	90 (79.6)
PSM on postoperative pathology, n	-	101
PSM with ECE, n (%)	-	68 (67.3)
PSM without ECE, n (%)	-	33 (32.7)
Pathologic stage	-	
pT2, n (%)	-	160 (58.6)
pT3, n (%)	-	113 (41.4)

Between the PCa patient group and the non-PCa patient group, there were significant differences in age, tPSA and f/tPSA (*P* < 0.05). Both between the ECE patient group and the non-ECE patient group and between the PSM patient group and the non-PSM patient group, there were significant differences in tPSA, positive core percentage and biopsyGS. The details are shown in Tables S1, S2, S3.

There were no obvious differences in clinical characteristics between the different training sets and corresponding testing sets. The characteristics of the included patients are summarized in Tables S4, S5, S6; the PCa patients constituted 59.4 and 59.6 % of the training and testing sets, respectively. The PCa patients with ECE constituted 41.7 and 40.7 % of the training and testing sets, respectively. The patients with a PSM constituted 37.0 % of both the training and testing sets. In the training and testing sets for benign and malignant prostate lesion discrimination, there were no significant differences in age, tPSA, or f/tPSA (*P* > 0.05; Table S7), and in the training and testing sets used for ECE and PSM prediction, there were no significant differences in tPSA, positive core percentage and biopsyGS (*P* > 0.05; Tables S8 and S9).

### Feature selection and acquisition of radiomics signatures

In total, 851 radiomic features per patient were extracted separately from the ROIs on the T2WI and ADC images. After removing features with low reproducibility or high redundancy, 278 T2WI features and 281 ADC features were retained for benign and malignant prostate lesion discrimination, 278 T2WI features and 293 ADC features were retained for ECE prediction, and 268 T2WI features and 295 ADC features were retained for PSM prediction. The remaining features were ranked by the MRMR approach, and the top 10 features were selected as the optimal radiomic feature subset.

The LASSO classifier was trained using the training set and the optimal radiomic feature subset to build the radiomics signatures. The details of the features used to build the different signatures are shown in Tables S10, S11, S12. These tables also show the positive and negative correlations and dependencies between the features and labels by providing the *p*-values and Spearman’s r-coefficients.

After the multivariable logistic regression analysis and AIC analysis, different combinations of ${S}_{T2WI}$, ${S}_{ADC}$, age, tPSA, f/tPSA, biopsyGS and positive core percentage were selected to build integrated models. The details and formulas are shown in Tables S13, S14, S15, S16, S17, S18, S19, S20, S21, S22, S23, S24, S25, S26, S27.

The performances of the radiomics signatures and integrated models of different characteristics are shown in Tables 2, 3 and 4. ${M}_{ADC}$ yielded the highest AUC of 0.912 for benign and malignant prostate lesion discrimination. ${M}_{ADC}$ achieved the highest AUC of 0.728 for ECE prediction.${M}_{ADC}$ yielded the highest AUC of 0.766 for PSM prediction. The *p-*values of the DeLong tests, 95 % CIs and the accuracy, sensitivity and specificity values are also shown in Tables 2, 3 and 4.

**Table 2:** The performance of radiomics signatures in the testing set for benign and malignant prostate lesion discrimination

	AUC	*p*-value	95 % CI	ACC	SEN	SPE	Cut-off
${S}_{T2WI}$	0.775	0.008	0.696, 0.842	0.699	0.654	0.782	0.622
${M}_{T2WI}$	0.851		0.780, 0.907	0.794	0.840	0.727	0.463
${S}_{ADC}$	0.863	0.024	0.793, 0.916	0.809	0.827	0.781	0.620
${M}_{ADC}$	0.912		0.852, 0.954	0.868	0.877	0.873	0.552
${S}_{MP-MRI}$	0.855	0.010	0.784, 0.909	0.801	0.815	0.782	0.574
${M}_{MP-MRI}$	0.905		0.843, 0.949	0.846	0.790	0.927	0.678

The *p*-values were derived from DeLong tests. The first row of *p*-values compares the AUCs of the radiomics signatures with random guesses by chance (AUC of 0.5), and the second row compares the AUCs of the radiomics signatures with those of the corresponding integrated models

**Table 3:** The performance of radiomics signatures in the testing set for ECE prediction

	AUC	*p*-value	95 % CI	ACC	SEN	SPE	Cut-off
${S}_{T2WI}$	0.599	0.002	0.484, 0.707	0.617	0.636	0.625	0.396
${M}_{T2WI}$	0.726		0.616, 0.819	0.691	0.849	0.583	0.267
${S}_{ADC}$	0.625	0.007	0.500, 0.724	0.580	0.697	0.521	0.386
${M}_{ADC}$	0.728		0.618, 0.821	0.691	0.727	0.688	0.427

**Table 4:** The performance of radiomics signatures in the testing set for PSM prediction

	AUC	*p*-value	95 % CI	ACC	SEN	SPE	Cut-off
${S}_{T2WI}$	0.614	0.099	0.499, 0.720	0.605	0.800	0.431	0.368
${M}_{T2WI}$	0.680		0.567, 0.779	0.691	0.633	0.745	0.414
${S}_{ADC}$	0.733	0.244	0.623, 0.826	0.667	0.867	0.569	0.311
${M}_{ADC}$	0.766		0.659, 0.853	0.728	0.667	0.765	0.360

**Fig. 3:**
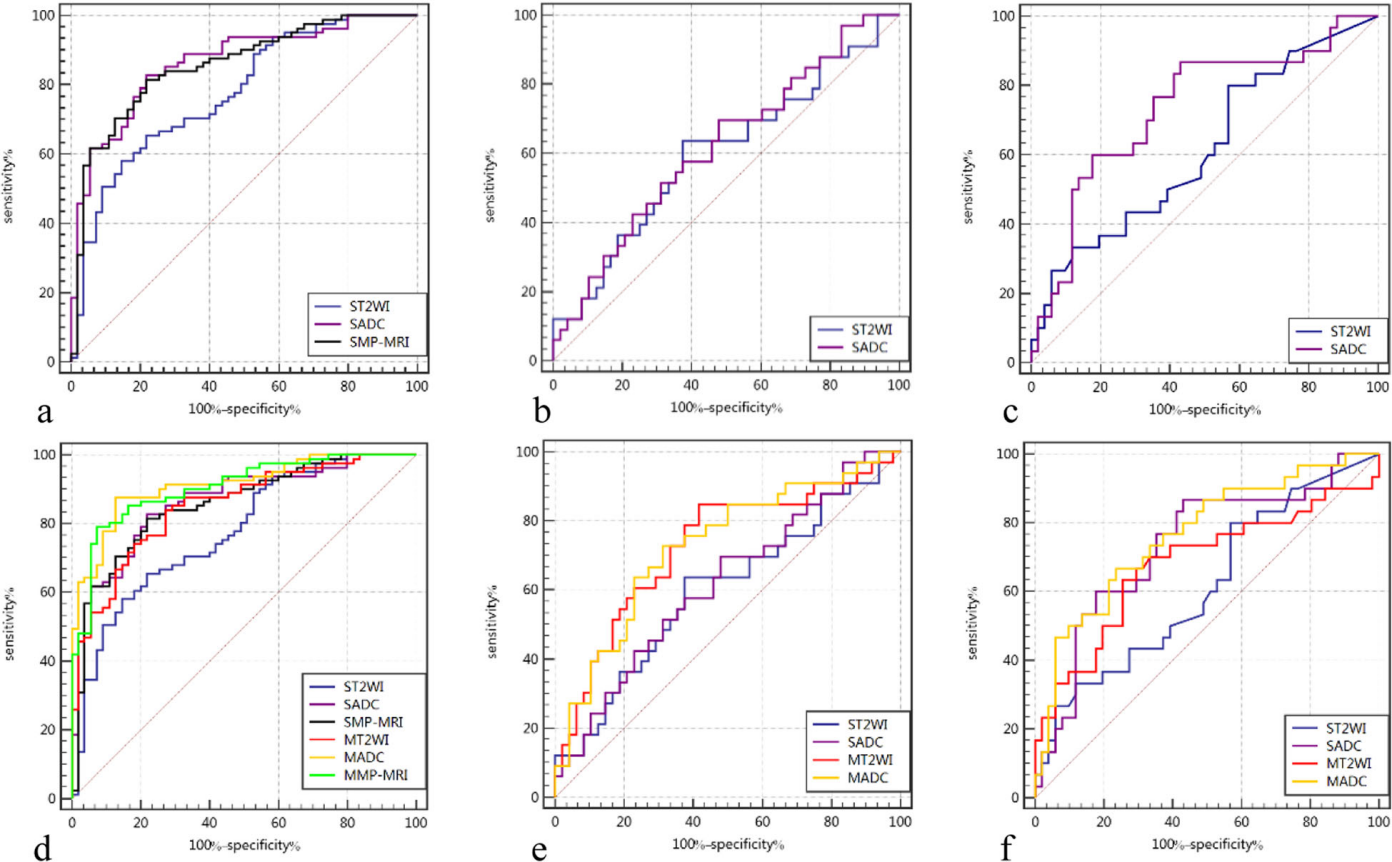
ROC curves of the radiomics signatures. **(a)** Radiomics signatures for benign and malignant prostate lesion discrimination. **(b)** Radiomics signatures for ECE prediction. **(c)** Radiomics signatures for PSM prediction. **(d)**, **(e)**, **(f)**. Combinations of the radiomics signatures and the integrated models for the three tasks separately to evaluate the effect of adding clinical information

The ROC curves of the different radiomics signatures, the integrated models and the combination of the radiomics signatures and integrated models for evaluating the effects of adding clinical information are shown in Fig. 3.

## Discussion

Despite the reliability of senior radiologists in detecting cancer lesions and boundaries, it is still challenging to determine the aggressiveness of PCa and predict the effect of surgical treatment. When estimating potential lesions, clinicians are limited to their personal knowledge and previous experience. These estimates are influenced by subjective judgements that may lead to variability among different clinicians. Machine learning based on the radiomics method can extract a large number of distinctive imaging features beyond those obtained by visual analysis by clinicians and build an objective prediction model. In our work, T2WI and ADC were used to build radiomics signatures for benign and malignant prostate lesion discrimination, ECE prediction and PSM prediction.

ADC and T2WI have been suggested to be negatively correlated with the percentage area of nuclei or cytoplasm measured in histopathological prostate tissue specimens and positively correlated with the percentage of luminal space [28]. With higher Gleason scores, a disrupted gland architecture results in a more chaotic and scattered appearance of the gland lumen [29]. The above findings were considered the histological basis of the correlations between PCa and the features on ADC and T2WI. In addition, ADC has been proven to provide more information than T2WI. In addition to providing information on cellularity, cell count and epithelial volume, ADC is inversely correlated with the expression of Ki-67, which is associated with proliferation potential [30].

The radiomic features ranked by the MRMR approach and then used in the radiomics signatures by LASSO were mostly wavelet-based features (8/10 for tumor discrimination, 3/6 for ECE prediction and 3/4 for PSM prediction). Wavelet-based features have a strong ability to predict labels and can quantify the heterogeneity of tumors at different scales that cannot be recognized by the naked eye [31]. These features play an important role in the establishment of radiomics signatures.

The radiomics signatures performed well in benign and malignant prostate lesion discrimination with the highest AUCs of 0.863 (ADC) and 0.775 (T2WI). The integrated model using the combination of the ADC signatures and clinical information reached an AUC of 0.912 and thus was better than the model combining the mpMRI signatures and clinical information. These findings suggest that the ADC sequence had more valid information for benign and malignant prostate lesion discrimination than the T2WI sequence. According to Table S4, age and f/tPSA were both included in the three integrated models. The clinical information and radiomic features were complementary and could be combined to obtain the prostate-related characteristics. Recent studies based on radiomics have used different features from different MRI sequences to discriminate benign prostate lesions from malignant lesions with AUCs ranging from 0.70 to 0.92 [21, 32]. Our findings are consistent with these studies, and we incorporated more clinical data from the patients for model optimization.

The radiomics signatures for ECE prediction achieved the highest AUC of 0.625 using the ADC sequence. Notably, among the 113 patients with ECE diagnosed by postoperative pathology, only 23 patients (20.3 %) were identified correctly by the preoperative MRI report with the manual identification method. There should be a correlation within in the radiomic features extracted from primary localized malignant lesions between the occurrence of ECE and tumor heterogeneity. The integrated models improved the performance to an AUC of 0.728 compared with the corresponding radiomics signatures with no clinical information. These results suggest that it is difficult to predict ECE using only preoperative MRI and that the inclusion of clinical information is necessary.

Previously, the factors used to predict PSM after RP in PCa mainly relied on clinical information. Turan et al. [33] identified that a positive core percentage of biopsy specimens, tPSA levels and the elapsed time between biopsy and surgery increased the risk of a PSM. Yang et al. [11] reported that perineural invasion, higher biopsy Gleason scores and more positive cores in biopsy specimens were independent predictors of a PSM. Although other predictors have been reported, such as body mass index, tPSA, prostate volume, and surgical experience, previous data have been largely inconclusive [34]. Considering the increasingly important role of mpMRI in the evaluation of PCa, there could be a potential association between radiomics and the occurrence of PSM. In this study, we selected patients whose operations were performed by the same senior urologist. As a result, the radiomics signatures performed well in the PSM prediction, with the highest AUC of 0.733 using ADC, and the corresponding intergraded model reached an AUC of 0.766. Radiomics signatures might reflect a number of characteristics. First, aggressive cancerous tissue blurs the boundary between normal and cancerous tissue under surgical vision, making it difficult to determine the boundary during surgery. Second, malignant tumor tissue loses stable tissue structure, increasing its susceptibility to invasion by surgical instruments compared with normal tissue during surgery.

Early studies have confirmed that age, tPSA and f/tPSA are significant parameters in PCa diagnosis [7]. The percentage of positive cores and the Gleason score based on biopsy pathology have been proven to be correlated with the aggressiveness of PCa [11, 35]. In our study, clinical data were fully utilized. A multivariable logistic regression analysis was conducted, and the AICs were compared to select risk factors from the above parameters for inclusion in different integrated models for optimization.

Prior studies have used machine learning models analyzing radiomic features to detect PCa and predict Gleason scores [16, 18, 21]. Some studies have demonstrated good correlations of mpMRI with other variables and ECE [36–38]. Alves et al. [39] achieved good results in an independent external validation of a nomogram using clinical data and imaging to predict ECE. Our study comprehensively analysed clinical variables and imaging features, and the results were consistent with previous studies. In addition, our experiments involved a relatively large amount of patient data, and we filtered potentially useful information from a larger number of possible features. Moreover, we explored the implied information contained in radiomic features in the prediction of ECE and PSM. We believe that applying our model could provide a reference for clinical judgment, thereby potentially improving diagnostic, prognostic, and predictive accuracy.

Our work has several limitations. First, the analysis was retrospectively performed using a dataset obtained at a single center. Further verification should be confirmed in a multi-center clinical study to estimate practicability. Second, we did not reanalyze the postoperative pathological slices but extracted information from pathology reports. Some uncertainty might exist in the match between the ROIs used to extract the radiomic features and the corresponding ROIs defined in the histological slices. Third, while DWI and dynamic contrast-enhanced MRI (DCE-MRI) have been proven to be accurate assessments of PCa, these methods were not included in our study because ADC is calculated from DWI; thus, there is an intrinsic connection between them, and the importance of DCE-MRI has diminished [40]. Furthermore, we did not apply radiomics assessment to lesions in different areas, such as peripheral and transitional zones and areas that were not well-defined.

In conclusion, our study demonstrates that radiomics signatures based on MRI could be used as a predictor in the discrimination of benign and malignant prostate lesions and the prediction of ECE and PSM. Prediction models were established, and we found that the combination of clinical data and radiomics signatures could improve the efficiency of the model compared to models including only radiomic features. Our findings might help clinicians better identify the risk of PCa compared with previous methods. Furthermore, wider tissue resection and more careful operation in PCa surgery should be considered when our model predicts risks of ECE and PSM.

## Supplementary Information

**Additional file 1:**

## Data Availability

The datasets used and/or analysed during the current study are available from the corresponding author on reasonable request.

## Abbreviations

ADC: Apparent diffusion coefficient
ECE: Extracapsular extension
LASSO: Least absolute shrinkage and selection operator
MP-MRI: Multiparametric MRI
MRMR: Minimum redundancy maximum relevance
PCa: Prostate cancer
ROI: Region of interest
T2WI: T2-weighted imaging
