# Supplementary material for: MRI-based radiomics models to assess prostate cancer, extracapsular extension and positive surgical margins
Source: Cancer Imaging. 2021 Jul 5;21:46. doi: 10.1186/s40644-021-00414-6 (PMC8259026; doi:10.1186/s40644-021-00414-6)
Supplement: Supplementary file 1 — Additional file 1: [file 40644_2021_414_MOESM1_ESM.docx]

**Supplementary materials**

**Table S1.** Clinical characteristics of the benign and malignant prostate lesion patient groups.

| Characteristics | Benign | Malignant | *p*-value |
| --- | --- | --- | --- |
| Number of patients | 186 | 273 |  |
| Age (y) | 65.23 ± 8.32 | 70.21 ± 6.70 | <0.001 |
| tPSA (ng/ml) | 13.63 ± 15.59 | 28.95 ± 33.07 | <0.001 |
| f/tPSA | 0.16 ± 0.77 | 0.13 ± 0.07 | <0.001 |

The *p*-values were derived from the independent t-test or the Mann–Whitney U test. SD, standard deviation.

**Table S2.** Clinical characteristics of the ECE and non-ECE patient groups.

|  | | Non-ECE | | ECE | *p*-value |
| --- | --- | --- | --- | --- | --- |
| Number of patients | | 160 | | 113 |  |
| Age (y) | | 70.34 ± 6.47 | | 70.04 ± 7.04 | 0.714 |
| tPSA (ng/ml) | | 19.40 ± 21.99 | | 42.47 ± 40.68 | <0.001 |
| f/tPSA | | 0.13 ± 0.07 | | 0.12 ± 0.06 | 0.053 |
| Positive core percentage | | 0.32 ± 0.22 | | 0.53 ± 0.30 | <0.001 |
| Biopsy GS | <7 | 123 |  | 49 | <0.001 |
|  | ≥7 | 37 |  | 64 |  |

**Table S3.** Clinical characteristics of the PSM and non-PSM patient groups.

| Characteristics | | Non-PSM | | PSM | *p*-value |
| --- | --- | --- | --- | --- | --- |
| Number of patients | | 172 | | 101 |  |
| Age (y) | | 70.27 ± 6.68 | | 70.11 ± 6.77 | 0.845 |
| tPSA (ng/ml) | | 24.37 ± 30.78 | | 36.75 ± 35.45 | 0.003 |
| f/tPSA | | 0.13 ± 0.07 | | 0.12 ± 0.07 | 0.078 |
| Positive core percentage | | 0.34 ± 0.24 | | 0.51 ± 0.30 | <0.001 |
| Biopsy GS | <7 | 126 |  | 46 | <0.001 |
|  | ≥7 | 46 |  | 55 |  |

**Table S4.** Clinical characteristics of the training and testing sets for benign and malignant prostate lesion discrimination.

| Characteristics | Training set | | *p*-value | Testing set | | *p*-value |
| --- | --- | --- | --- | --- | --- | --- |
|  | Benign | Malignant |  | Benign | Malignant |  |
| No. of patients | 131 | 192 |  | 55 | 81 |  |
| Age (y)  (mean±SD) | 62.33 ± 7.61 | 70.62 ± 6.33 | <0.001 | 65.00 ± 9.89 | 69.25 ± 7.46 | 0.005 |
| tPSA (ng/mL) (mean±SD) | 13.68 ± 17.32 | 29.46 ± 39.72 | <0.001 | 13.50 ± 10.54 | 27.73 ± 31.64 | <0.001 |
| f/tPSA  (mean±SD) | 0.16 ± 0.08 | 0.12 ± 0.07 | <0.001 | 0.17 ± 0.07 | 0.13 ± 0.07 | 0.001 |

The *p*-values were derived from independent t-tests or Mann-Whitney U tests. SD, standard deviation.

**Table S5.** Clinical characteristics of the training and testing sets for ECE prediction.

| Characteristics | | Training set |  | *p*-value | Testing set |  | *p*-value |
| --- | --- | --- | --- | --- | --- | --- | --- |
|  | | Non-ECE | ECE |  | Non-ECE | ECE |  |
| Number of patients | | 112 | 80 |  | 48 | 33 |  |
| Age (y)  (mean±SD) | | 70.57 ± 6.06 | 70.16 ± 6.45 | 0.521 | 69.37 ± 7.24 | 69.72 ± 8.41 | 0.840 |
| tPSA (ng/mL) (mean±SD) | | 20.77 ± 24.24 | 43.41 ± 43.83 | <0.001 | 16.21 ± 15.30 | 40.16 ± 32.26 | <0.001 |
| f/tPSA  (mean±SD) | | 0.13 ± 0.07 | 0.12 ± 0.07 | 0.200 | 0.14 ± 0.07 | 0.11 ± 0.06 | 0.112 |
| Positive core percentage  (mean±SD) | | 0.33 ± 0.23 | 0.51 ± 0.29 | <0.001 | 0.30 ± 0.20 | 0.59 ± 0.32 | <0.001 |
| BiopsyGS | <7 | 87 | 35 | <0.001 | 36 | 14 | 0.003 |
|  | ≥7 | 25 | 45 |  | 12 | 19 |  |

**Table S6.** Clinical characteristics of the training and testing sets for PSM prediction.

| Characteristics | | Training set |  | *p*-value | Testing set | |  | *p*-value |
| --- | --- | --- | --- | --- | --- | --- | --- | --- |
|  | | Non-PSM | PSM |  | Non-PSM | | PSM |  |
| Number of patients | | 121 | 71 |  | 51 | | 30 |  |
| Age (y)  (mean±SD) | | 70.41 ± 6.35 | 70.65 ± 6.47 | 0.806 | 69.94 ± 7.46 | | 68.83 ± 7.39 | 0.519 |
| tPSA (ng/mL)  (mean±SD) | | 25.31 ± 32.90 | 37.10 ± 35.75 | 0.021 | 22.14 ± 25.23 | | 35.90 ± 35.30 | 0.045 |
| f/tPSA  (mean±SD) | | 0.14 ± 0.08 | 0.12 ± 0.07 | 0.236 | 0.12 ± 0.06 | | 0.10 ± 0.04 | 0.098 |
| Positive core percentage (mean±SD) | | 0.33 ± 0.23 | 0.51 ± 0.29 | <0.001 | 0.37 ± 0.27 | | 0.54 ± 0.32 | 0.014 |
| BiopsyGS | <7 | 90 | 32 | <0.001 | 36 | 14 | | 0.032 |
|  | ≥7 | 31 | 39 |  | 15 | 16 | |  |

**Table S7.** Selected clinical characteristics of the training and testing sets for benign and malignant prostate lesion discrimination.

| Characteristics | Training set | Testing set | *p*-value |
| --- | --- | --- | --- |
|  |  |  |  |
| Age (y)  (mean±SD) | 67.47 ± 7.34 | 67.53 ± 8.75 | 0.236 |
| tPSA (ng/mL)  (mean±SD) | 23.06 ± 29.26 | 21.98 ± 26.21 | 0.709 |
| f/tPSA  (mean±SD) | 0.14 ± 0.08 | 0.14 ± 0.07 | 0.976 |

The *p*-values were derived from the independent t-test. SD, standard deviation.

**Table S8.** Selected clinical characteristics of the training and testing sets for ECE prediction.

| Characteristics | | Training set |  | Testing set | *p*-value |
| --- | --- | --- | --- | --- | --- |
|  | |  |  |  |  |
| tPSA  (mean±SD) | | 30.21 ± 35.52 |  | 25.97 ± 26.35 | 0.335 |
| Positive core percentage  (mean±SD) | | 0.40 ± 0.27 |  | 0.42 ± 0.29 | 0.571 |
| Biopsy GS | <7 | 122 |  | 50 | 0.777 |
|  | ≥7 | 70 |  | 31 |  |

**Table S9.** Selected clinical characteristics of the training and testing sets for PSM prediction.

| Characteristics | | Training set | Testing set | *p*-value |
| --- | --- | --- | --- | --- |
| (mean±SD) | |  |  |  |
| tPSA  (mean±SD) | | 29.67±34.36 | 27.24±29.91 | 0.580 |
| Positive core percentage  (mean±SD) | | 0.40±0.27 | 0.43±0.30 | 0.335 |
| Biopsy GS | <7 | 122 | 50 | 0.777 |
|  | ≥7 | 70 | 31 |  |

**Table S10.** The selected features of the radiomics signatures for benign and malignant lesion discrimination.

|  | Selected features | *r* | *p*-value |
| --- | --- | --- | --- |
| $S_{T2WI}$ | original_shape_Maximum2DDiameterColumn | 0.314 | <0.001 |
|  | wavelet.LLH_firstorder_Maximum | 0.268 | <0.001 |
|  | wavelet.LLH_gldm_LargeDependenceLowGrayLevelEmphasis | 0.329 | <0.001 |
|  | wavelet.LHL_firstorder_Skewness | 0.233 | <0.001 |
|  | wavelet.LHH_glcm_SumAverage | 0.145 | <0.001 |
|  | wavelet.HLL_glcm_Imc2 | -0.261 | <0.001 |
|  | wavelet.HLL_glszm_SizeZoneNonUniformityNormalized | 0.206 | <0.001 |
|  | wavelet.HHH_ngtdm_Complexity | 0.355 | <0.001 |
|  | wavelet.LLL_gldm_DependenceNonUniformityNormalized | -0.262 | <0.001 |
| $S_{ADC}$ | original_firstorder_InterquartileRange | 0.262 | <0.001 |
|  | original_firstorder_Minimum | -0.402 | <0.001 |
|  | wavelet.LLH_firstorder_TotalEnergy | 0.244 | <0.001 |
|  | wavelet.LHL_glszm_SmallAreaLowGrayLevelEmphasis | 0.283 | <0.001 |
|  | wavelet.LHL_gldm_LargeDependenceHighGrayLevelEmphasis | 0.438 | <0.001 |
|  | wavelet.HLL_glcm_ClusterShade | -0.212 | <0.001 |
|  | wavelet.HLH_glszm_SmallAreaHighGrayLevelEmphasis | 0.220 | <0.001 |
|  | wavelet.HLH_ngtdm_Complexity | 0.283 | <0.001 |
|  | wavelet.LLL_glrlm_LongRunEmphasis | -0.200 | <0.001 |

**Table S11.** The selected features of the radiomics signatures for ECE prediction.

|  | Selected features | *r* | *p*-value |
| --- | --- | --- | --- |
| $S_{T2WI}$ | original_glszm_SizeZoneNonUniformityNormalized | -0.181 | 0.012 |
|  | wavelet.LLH_glszm_GrayLevelNonUniformity | 0.305 | <0.001 |
|  | wavelet.LHH_firstorder_Mean | -0.286 | <0.001 |
|  | wavelet.LLL_glszm_ZoneVariance | 0.334 | <0.001 |
| $S_{ADC}$ | original_shape_MeshVolume | 0.291 | <0.001 |
|  | original_firstorder_10Percentile | -0.286 | <0.001 |
|  | original_glszm_SizeZoneNonUniformity | 0.305 | <0.001 |
|  | wavelet.LHH_glszm_SizeZoneNonUniformity | 0.263 | <0.001 |
|  | wavelet.HLH_gldm_DependenceNonUniformityNormalized | -0.325 | <0.001 |
|  | wavelet.LLL_glszm_LowGrayLevelZoneEmphasis | -0.285 | <0.001 |

**Table S12.** The selected features of the radiomics signatures for PSM prediction.

|  | Selected features | *r* | *p*-value |
| --- | --- | --- | --- |
| $S_{T2WI}$ | wavelet.HHL_glszm_SizeZoneNonUniformity | 0.331 | <0.001 |
| $S_{ADC}$ | original_glszm_SmallAreaHighGrayLevelEmphasis | 0.244 | <0.001 |
|  | wavelet.LLH_glszm_SizeZoneNonUniformity | 0.291 | <0.001 |
|  | wavelet.LLL_firstorder_Skewness | 0.265 | <0.001 |
|  | wavelet.LLL_glszm_HighGrayLevelZoneEmphasis | 0.323 | <0.001 |

**Table S13.** Multivariable logistic regression analysis results for $S_{MP-MRI}$ for benign and malignant prostate lesion discrimination.

| Variable | Final model |  |
| --- | --- | --- |
|  | β | *p*-value |
| Intercept | -6.0185 | <0.001 |
| $S_{T2WI}$ | 5.14 | 0.002 |
| $S_{ADC}$ | 5.624 | <0.001 |

**Table S14.** Multivariable logistic regression analysis results for $M_{T2WI}$ for benign and malignant prostate lesion discrimination.

| Variable | Initial model |  |  | Final model |  |
| --- | --- | --- | --- | --- | --- |
|  | β | *p*-value |  | β | *p*-value |
| Intercept | -11.428 | <0.001 |  | -11.83 | <0.001 |
| $S_{T2WI}$ | 8.888 | <0.001 |  | 9.415 | <0.001 |
| Age | 0.109 | <0.001 |  | 0.115 | <0.001 |
| tPSA | 0.013 | 0.087 |  | NA | NA |
| f/tPSA | -8.294 | <0.001 |  | -8.747 | <0.001 |

**Table S15.** Multivariable logistic regression analysis results for $M_{T2WI}$ for benign and malignant prostate lesion discrimination.

| Variable | Final model |  |
| --- | --- | --- |
|  | β | *p*-value |
| Intercept | -11.37 | <0.001 |
| $S_{ADC}$ | 6.925 | <0.001 |
| Age | 0.123 | <0.001 |
| tPSA | 0.016 | 0.037 |
| f/tPSA | -6.749 | 0.002 |

**Table S16.** Multivariable logistic regression analysis results for $M_{MP-MRI}$ for benign and malignant prostate lesion discrimination.

|  | Initial model |  |  | Final model |  |
| --- | --- | --- | --- | --- | --- |
| Variable | β | *p*-value |  | β | *p*-value |
| Intercept | -12.355 | <0.001 |  | -12.63 | <0.001 |
| $S_{T2WI}$ | 3.658 | 0.041 |  | 4.277 | 0.014 |
| $S_{ADC}$ | 5.665 | <0.001 |  | 5.636 | <0.001 |
| Age | 0.117 | <0.001 |  | 0.121 | <0.001 |
| tPSA | 0.013 | 0.096 |  | NA | NA |
| f/tPSA | -6.855 | 0.002 |  | -7.421 | <0.001 |

**Table S17.** Multivariable logistic regression analysis results for $S_{MP-MRI}$ for ECE prediction.

| Variable | Final model |  |
| --- | --- | --- |
|  | β | *p*-value |
| Intercept | -4.681 | <0.001 |
| $S_{T2WI}$ | 3.533 | 0.07 |
| $S_{ADC}$ | 6.899 | 0.001 |

**Table S18.** Multivariable logistic regression analysis results for $M_{T2WI}$ for ECE prediction.

| Variable | Initial model |  | |  | | Final model |  |
| --- | --- | --- | --- | --- | --- | --- | --- |
|  | β | *p*-value | |  | | β | *p*-value |
| Intercept | -4.529 | <0.001 |  | | | -4.64 | <0.001 |
| $S_{T2WI}$ | 7.26 | <0.001 | | |  | 7.637 | <0.001 |
| tPSA | 0.007 | 0.244 | | |  | NA | NA |
| BiopsyGS (1 vs. 0) | 0.989 | 0.009 | | |  | 1.048 | 0.005 |
| Positive core percentage | 1.432 | 0.06 | | |  | 1.804 | 0.011 |

**Table S19.** Multivariable logistic regression analysis results for $M_{T2WI}$ for ECE prediction.

| Variable | Initial model |  | |  | Final model |  |
| --- | --- | --- | --- | --- | --- | --- |
|  | β | *p*-value | |  | β | *p*-value |
| Intercept | -5.177 | <0.001 |  | | -5.276 | <0.001 |
| $S_{ADC}$ | 8.986 | <0.001 |  | | 9.286 | <0.001 |
| tPSA | 0.004 | 0.569 |  | | NA | NA |
| BiopsyGS (1 vs. 0) | 1.128 | 0.004 |  | | 1.167 | 0.002 |
| Positive core percentage | 1.399 | 0.071 |  | | 1.573 | 0.028 |

**Table S20.** Multivariable logistic regression analysis results for $M_{MP-MRI}$ for ECE prediction.

|  | Initial model |  |  | Final model |  |
| --- | --- | --- | --- | --- | --- |
| Variable | β | *p*-value |  | β | *p*-value |
| Intercept | -5.542 | <0.001 |  | -5.276 | <0.001 |
| $S_{T2WI}$ | 3.277 | 0.107 |  | NA | NA |
| $S_{ADC}$ | 6.625 | 0.004 |  | 9.286 | <0.001 |
| tPSA | 0.003 | 0.581 |  | NA | NA |
| BiopsyGS (1 vs. 0) | 1.094 | 0.005 |  | 1.167 | 0.002 |
| Positive core percentage | 1.446 | 0.068 |  | 1.573 | 0.028 |

**Table S21.** Multivariable logistic regression analysis results for $S_{MP-MRI}$ for PSM prediction.

| Variable | Final model |  |
| --- | --- | --- |
|  | β | *p*-value |
| Intercept | -3.079 | 0.354 |
| $S_{T2WI}$ | -2.000 | 0.839 |
| $S_{ADC}$ | 8.655 | <0.001 |

**Table S22.** Multivariable logistic regression analysis results for $M_{T2WI}$ for PSM prediction.

| Variable | Initial model |  | |  | Final model |  |
| --- | --- | --- | --- | --- | --- | --- |
|  | β | *p*-value | |  | β | *p*-value |
| Intercept | -12.501 | <0.001 |  | | -12.314 | <0.001 |
| $S_{T2WI}$ | 28.183 | 0.001 |  | | 28.624 | 0.001 |
| tPSA | -0.003 | 0.6 |  | | NA | NA |
| BiopsyGS (1 vs. 0) | 0.856 | 0.016 |  | | 0.829 | 0.018 |
| Positive core percentage | 2.286 | 0.001 |  | | 2.154 | 0.001 |

**Table S23.** Multivariable logistic regression analysis results for $M_{T2WI}$ for PSM prediction.

| Variable | Initial model |  | |  | Final model |  |
| --- | --- | --- | --- | --- | --- | --- |
|  | β | *p*-value | |  | β | *p*-value |
| Intercept | -4.837 | <0.001 |  | | -4.723 | <0.001 |
| $S_{ADC}$ | 8.589 | <0.001 |  | | 8.168 | <0.001 |
| tPSA | -0.007 | 0.205 |  | | NA | NA |
| BiopsyGS (1 vs. 0) | 0.989 | 0.011 |  | | 0.909 | 0.017 |
| Positive core percentage | 2.186 | 0.005 |  | | 1.798 | 0.013 |

**Table S24.** Multivariable logistic regression analysis results for $M_{MP-MRI}$ for PSM prediction.

|  | Initial model |  |  | Final model |  |
| --- | --- | --- | --- | --- | --- |
| Variable | β | *p*-value |  | β | *p*-value |
| Intercept | -4.285 | 0.236 |  | -4.723 | <0.001 |
| $S_{T2WI}$ | -1.642 | 0.877 |  | NA | NA |
| $S_{ADC}$ | 8.738 | <0.001 |  | 8.168 | <0.001 |
| tPSA | -0.007 | 0.206 |  | NA | NA |
| BiopsyGS (1 vs. 0) | 0.996 | 0.011 |  | 0.909 | 0.017 |
| Positive core percentage | 2.174 | 0.006 |  | 1.798 | 0.013 |

**Table S25.** The formulas of the integrated model for benign and malignant prostate lesion discrimination.

|  | Formulas |
| --- | --- |
| $S_{MP-MRI}$ | -6.019+5.14$\times S_{T2WI}$+ 5.624$\times S_{ADC}$ |
| $M_{T2WI}$ | -11.836+9.415$\times S_{T2WI}$+0.115$\times$age-8.747$\times$f/tPSA |
| $M_{ADC}$ | -11.37+6.925$\times S_{ADC}$+0.123$\times$age +0.016$\times$tPSA -6.749/tPSA |
| $M_{MP-MRI}$ | -12.63 + 4.277$\times S_{T2WI}$+ 5.636$\times S_{ADC}$+0.121$\times$age-7.422f/tPSA |

**Table S26.** The formulas of the integrated model for ECE prediction.

|  | Formulas |
| --- | --- |
| $M_{T2WI}$ | -4.64+7.637$\times S_{T2WI}$ +1.048$\times$biopsyGS  +1.804$\times$positive core percentage |
| $M_{ADC}$ | -5.276+9.286$\times S_{ADC}$+1.167$\times$biopsyGS  +1.573$\times$positive core percentage |

**Table S27** The formulas of the integrated model for PSM prediction

|  | Formulas |
| --- | --- |
| $M_{T2WI}$ | -12.314+28.624$\times S_{T2WI}$+0.829$\times$biopsyGS  +2.15$\times$positive core percentage |
| $M_{ADC}$ | -4.723+8.168$\times S_{ADC}$+0.909$\times$biopsyGS  +1.798$\times$positive core percentage |

STARD checklist

|  | **Section & Topic** | **No** | **Item** |  | **page** |
| --- | --- | --- | --- | --- | --- |
|  |  |  |  |  |  |
|  | **TITLE OR ABSTRACT** |  |  |  |  |
|  |  | **1** | Identification as a study of diagnostic accuracy using at least one measure of accuracy  (such as sensitivity, specificity, predictive values, or AUC) | √ | 1 |
|  | **ABSTRACT** |  |  |  |  |
|  |  | **2** | Structured summary of study design, methods, results, and conclusions  (for specific guidance, see STARD for Abstracts) | √ | 2 |
|  | **INTRODUCTION** |  |  |  |  |
|  |  | **3** | Scientific and clinical background, including the intended use and clinical role of the index test | √ | 4 |
|  |  | **4** | Study objectives and hypotheses | √ | 4-5 |
|  | **METHODS** |  |  |  |  |
|  | *Study design* | **5** | Whether data collection was planned before the index test and reference standard  were performed (prospective study) or after (retrospective study) | √ | 5-6 |
|  | *Participants* | **6** | Eligibility criteria | √ | 6 |
|  |  | **7** | On what basis potentially eligible participants were identified  (such as symptoms, results from previous tests, inclusion in registry) | √ | 6 |
|  |  | **8** | Where and when potentially eligible participants were identified (setting, location and dates) | √ | 6 |
|  |  | **9** | Whether participants formed a consecutive, random or convenience series | √ | 6 |
|  | *Test methods* | **10a** | Index test, in sufficient detail to allow replication | × | - |
|  |  | **10b** | Reference standard, in sufficient detail to allow replication | √ | 6 |
|  |  | **11** | Rationale for choosing the reference standard (if alternatives exist) | √ | 6 |
|  |  | **12a** | Definition of and rationale for test positivity cut-offs or result categories  of the index test, distinguishing pre-specified from exploratory | × | - |
|  |  | **12b** | Definition of and rationale for test positivity cut-offs or result categories  of the reference standard, distinguishing pre-specified from exploratory | √ | 6-12 |
|  |  | **13a** | Whether clinical information and reference standard results were available  to the performers/readers of the index test | √ | 6-9 |
|  |  | **13b** | Whether clinical information and index test results were available  to the assessors of the reference standard | × |  |
|  | *Analysis* | **14** | Methods for estimating or comparing measures of diagnostic accuracy | √ | 6 |
|  |  | **15** | How indeterminate index test or reference standard results were handled | √ | 6-12 |
|  |  | **16** | How missing data on the index test and reference standard were handled | √ | 6 |
|  |  | **17** | Any analyses of variability in diagnostic accuracy, distinguishing pre-specified from exploratory | √ | 12 |
|  |  | **18** | Intended sample size and how it was determined | √ | 9 |
|  | **RESULTS** |  |  |  |  |
|  | *Participants* | **19** | Flow of participants, using a diagram | √ | Table 1&Figure 1 |
|  |  | **20** | Baseline demographic and clinical characteristics of participants | √ | Table 1 |
|  |  | **21a** | Distribution of severity of disease in those with the target condition | √ | Table 1 |
|  |  | **21b** | Distribution of alternative diagnoses in those without the target condition | √ | Table 1 |
|  |  | **22** | Time interval and any clinical interventions between index test and reference standard | √ | 6 |
|  | *Test results* | **23** | Cross tabulation of the index test results (or their distribution)  by the results of the reference standard |  |  |
|  |  | **24** | Estimates of diagnostic accuracy and their precision (such as 95% confidence intervals) | √ | Table 2-4 |
|  |  | **25** | Any adverse events from performing the index test or the reference standard | × | - |
|  | **DISCUSSION** |  |  |  |  |
|  |  | **26** | Study limitations, including sources of potential bias, statistical uncertainty, and generalisability | √ | 17 |
|  |  | **27** | Implications for practice, including the intended use and clinical role of the index test | √ | 16 |
|  | **OTHER INFORMATION** |  |  |  |  |
|  |  | **28** | Registration number and name of registry | √ | 18 |
|  |  | **29** | Where the full study protocol can be accessed | √ | 6-10 |
|  |  | **30** | Sources of funding and other support; role of funders | √ | 18 |
|  |  |  |  |  |  |
